# Vagus nerve stimulation and inflammation: expanding the scope beyond cytokines

**DOI:** 10.1186/s42234-022-00100-3

**Published:** 2022-12-01

**Authors:** Aidan Falvey

**Affiliations:** Institute of Bioelectronic Medicine, The Feinstein Institutes for Medical Research, Northwell Health, 350 Community Drive, 11030 Manhasset, NY USA

**Keywords:** Cytokines, Inflammation, Inflammatory reflex, Neutrophils, Resolution, Specialized pro-resolving mediators, Vagus nerve stimulation

## Abstract

Approximately 20 years ago it was discovered that the vagus nerve regulates pro-inflammatory cytokine levels and inflammation. Subsequent research using several preclinical models revealed that vagus nerve stimulation evokes a protective decrease in pro-inflammatory cytokines in multiple inflammatory disorders. Consequently, the pro- and anti- inflammatory cytokine balance has become the predominant readout for indicating a positive outcome of vagus nerve stimulation. However, cytokine levels are just a single aspect of an effective immune response. It is conceivable that vagus nerve stimulation regulates inflammation through additional mechanisms. In this letter, I discuss a manuscript that describes how vagus nerve stimulation promotes resolution of inflammation via regulating the balance of specialised pro-resolving mediator levels and neutrophil activity.

## Main text

For a long time, the dogmatic point of view was that the nervous system and the immune system were anatomically and functionally distinct. It is now evident that these systems are synergistically intertwined forming a neuro-immune dialogue throughout the body. Instrumental to this discovery was the seminal paper by Borovikova [2000] et al. – V*agus nerve stimulation attenuates the systemic inflammatory response to endotoxin *– from the laboratory of Dr. Kevin J. Tracey (Borovikova et al. 2000). This manuscript was the first to demonstrate that the vagus nerve regulates systemic cytokine levels such as tumour necrosis factor (TNF) and that electrical stimulation of the vagus nerve (VNS) reduces endotoxin-induced cytokine levels. These findings demonstrated the critical relationship between the nervous and immune system in the regulation of inflammation and ultimately led to launching the field of bioelectronic medicine.

Upon an immune challenge, inflammation is triggered in the form of recruited and activated leukocytes and their important mediators – cytokines. Cytokines, including TNF, are a large group of signaling molecules crucial to the inflammatory response with independent and synergistic effects that can mediate pro- or anti-inflammatory effects. Further, the balance of pro- and anti-inflammatory cytokines is essential: in certain conditions (such as inflammatory bowel disease (IBD) and rheumatoid arthritis) the balance shifts towards a chronic pro-inflammatory environment – mediating inflammation and cell death.

A vagus nerve-mediated physiological mechanism termed *the inflammatory reflex*regulates the levels of pro-inflammatory cytokines and inflammation (Tracey 2002). The efferent arm of the inflammatory reflex begins in a brainstem nucleus - the dorsal motor nucleus of the vagus (DMN) (Kressel et al. 2020). Neurons extend from this nucleus along the vagus nerve to the celiac superior mesenteric ganglion complex where they interact with the splenic nerve. The splenic nerve releases norepinephrine in the spleen parenchyma, which triggers the release of acetylcholine (ACh) from a subset of T-cells expressing the enzyme choline acetyltransferase (i.e. ChAT T-cells). ACh then acts via α7 nicotinic ACh receptors (α7nAChR) expressed on macrophages to suppress the production and release of TNF and other pro-inflammatory cytokines. This endogenous reflex can also be amplified by external stimuli such as electrical stimulation, which has shown success in preclinical models of multiple inflammatory conditions (Falvey et al. 2022; Pavlov and Tracey 2022). Further, this knowledge has been translated to successful clinical trials utilizing electrical/bioelectronic VNS in patients with IBD and rheumatoid arthritis (Bonaz et al. 2016; Koopman et al. 2016). This approach was first assessed and validated in these disorders due to the pronounced effect of VNS on the serum levels of TNF - a key molecular mediator of inflammation and disease pathogenesis (Falvey et al. 2022; Pavlov and Tracey 2022). Preclinical research investigating the efficacy of VNS in controlling inflammation has focused on the cytokine response to stimulation. However, cytokines are a single, albeit important, component of the immune response and it is conceivable that VNS has a much broader role in regulating the immune system. That is why I was excited to read, and I highlight in this letter, a recent manuscript by Caravaca et al.,- *Vagus nerve stimulation promotes resolution of inflammation by a mechanism that involves Alox15 and requires the α7nAChR subunit *(Caravaca et al. 2022) recently published by Dr. Peder Olofsson’s team, which expands the known boundaries of immune regulation by VNS.

In this manuscript, Dr. Olofsson and his team describe how VNS influences the resolution of inflammation. Using a model of zymosan-induced peritonitis, they demonstrate that VNS significantly shortened the time to inflammation resolution, promoted both efferocytosis and a pro-resolving lipid profile via the α7nAChR. This effect was also partially dependent upon the 12/15-lipoxygenase (ALOX 15) – a key enzyme in the biosynthesis of specialized pro-resolving mediators (SPMs). The most common leukocytes in circulation are neutrophils, which are granulocytes with a short half-life. Neutrophils are among the first leukocytes recruited to a site of infection. Their primary role is to release ‘neutrophil extracellular traps’ or NETs, which are comprised of their own DNA and extracellular matrix that serve to trap invading pathogens that are subsequently engulfed by mononuclear phagocytes (i.e. macrophages). In the resolution of inflammation, these exhausted neutrophils are also engulfed by macrophages in a process known as efferocytosis. Neutrophil number and activity are indicators of inflammation, their reduction in number over time, as well as the speed by which they are cleared, are indicators of resolution of inflammation. Caravaca and co-authors performed VNS one hour prior to zymosan intraperitoneal injection and collected peritoneal exudate at 4, 12, 24, and 48 hours post-injection. Flow cytometry was used to quantify the total number of neutrophils which were plotted and modelled using an ordinary differential equation (ODE) model. A typical variable that can be calculated from this model is the resolution index (R_i_), which is the calculated time from peak neutrophil number to 50% of that count. It was shown that VNS significantly reduced the R_i_ of neutrophils in peritoneal exudate when compared to sham mice. As the peak neutrophil number may not be known, the authors utilised an additional statistical tool to describe their data curves – inflammation decay (I_d_). This index indicates neutrophil decay, and it can be calculated without the knowledge of peak neutrophil number. I_d_ is based on the author’s observation that the decay in neutrophil number appears to be exponential within the peritoneal cavity over time. The authors found that VNS induced a higher rate of I_d_ when compared to sham mice. Together, these results indicate that resolution of inflammation occurred faster post-VNS Vs. sham mice. Furthermore, macrophage efferocytosis of neutrophils, when assessed 12 hours post zymosan injection, was significantly higher in VNS-treated mice when compared to sham mice.

Interestingly, the authors also performed the first analysis, *in-vivo*, of SPMs post VNS. SPMs are a large class of lipid signaling molecules that contribute to the resolution of the inflammation by counter regulating pro-inflammatory cytokines and promoting macrophage efferocytosis. While greater clarification on their mechanism of action is required, the activity of many of these molecules indicates them to be neuroprotective and overall contribute to the resolution of inflammation. The authors performed VNS as before, injected zymosan, and then collected peritoneal exudates 12 h later for analysis by liquid-chromatography-tandem mass spectrometry-based metabololipidomics. VNS-mice, compared to sham mice, was positively associated with the greater concentration in omega-3 docosahexaenoic (DHA) and docosapentaenoic acid (n-3 DPA)-derived SPM families – specifically the D series resolvins (RvD), protectins (PD), maresins (MaR) derived from DHA and PD derived from n-3 DPA. Further, the authors demonstrated that the prostaglandin (pro-inflammatory lipid signaling molecule) to SPM ratio was reduced in VNS-treated mice. Together these results indicate a shift from a pro-inflammatory to a pro-resolving phenotype in VNS-treated mice.

A reduction in pro-inflammatory cytokines is a solid indicator of an effective interventional therapeutic for alleviating aberrant inflammation. This readout has become the gold-standard in the field of bioelectronic medicine and it has been instrumental in translating preclinical research to the clinic. Recently, many new neuro-immune pathways have been discovered (Falvey et al. 2022; Ahmed et al. 2022). These pathways have predominantly focused on developing an intervention that alters the pro- and anti-inflammatory cytokine balance. Caravaca et al. clearly demonstrate the broader scope of VNS applicability in treating inflammation beyond alterations in cytokine levels. The authors’ findings indicate new immunological-based readouts that can be considered in future studies with VNS and other bioelectronic medicine approaches with the ultimate goal to provide therapeutic benefit in numerous inflammatory diseases. 

## Data Availability

Not applicable.

## Abbreviations

12/15 Lipoxygenase: (ALOX 15)
α7nAChR: Alpha 7 nicotinic acetylcholine receptor
ACh: Acetycholine
ChAT: Choline acetyltransferae
DHA: docosahexaenoic acid (omega 3)
DMN: Dorsal motor nucleus of the vagus
IBD: Inflammatory bowel disease
I_D_: Inflammation decay
MaR: Maresins
n-3 DPA: n-3 docosapentaenoic acid
ODE: Ordinary differential equation
PD: Protectins
R_i_: Resolution index
RvD: D Series Resolvins
SPM: Specialized pro-resolving mediators
TNF: Tumour necrosis factor
VNS: Vagus nerve stimulation
